# Influence of combined posterior and medial-lateral mid-air trunk perturbations on knee biomechanics during single-leg landing

**DOI:** 10.3389/fspor.2025.1697893

**Published:** 2025-11-03

**Authors:** Yu Song, Wanyan Su, Yu Gu, Nawfal Malik, Thanh Nguyen, Anne Jordan, Elijah Savala, Boyi Dai

**Affiliations:** ^1^ Department of Health, Sport and Exercise Sciences, University of Kansas, Lawrence, KS, United States; ^2^ College of Education and Health Sciences, Haskell Indian Nations University, Lawrence, KS, United States; ^3^ Department of Rehabilitation and Movement Science, University of Vermont, Burlington, VT, United States

**Keywords:** anterior cruciate ligament, ACL, kinematics, kinetics, ACL injury

## Abstract

**Objectives:**

To determine the effect of combined posterior and medial-lateral mid-air trunk perturbation on biomechanical variables associated with ACL loading during single-leg landings.

**Design:**

Controlled laboratory investigation with a repeated-measures design.

**Method:**

Thirty-seven injury-free reactional athletes performed double-leg jump and single-leg landing tasks under three mid-air trunk pulling perturbation conditions (posterior-lateral, posterior-medial, and no perturbation relative to the landing leg). Kinematic and ground reaction force (GRF) data were collected. Jump height, trunk flexion and lateral bending angles, and knee angles and moments during landing were calculated. Paired *t*-tests were performed to assess perturbation consistencies, while one-by-three repeated-measures ANOVAs were applied to other variables (α = 0.05).

**Results:**

No significant differences were observed in perturbation duration, timing, and jump height (*p* ≥ 0.276). Posterior-lateral perturbation demonstrated the greatest trunk lateral bending angles, knee flexion angle at initial ground contact (IC), peak knee abduction/internal rotation angles, peak posterior GRF, and peak knee adduction moments during landing compared to other conditions (*p* ≤ 0.004). Posterior-medial perturbation showed the smallest trunk flexion angles and knee flexion angles among all conditions (*p* ≤ 0.035), while greater peak posterior GRF and knee extension moments compared to no perturbation (*p* < 0.001).

**Conclusions:**

Posterior-lateral perturbation resulted in increased trunk lateral bending, leading to increased ACL loading variables in the frontal plane during single-leg landing. Additionally, posterior-medial perturbation primarily increased sagittal plane ACL loading variables. These findings help understand indirect-contact ACL injury mechanisms and highlight the importance of optimizing trunk control strategies in injury prevention.

## 1 Introduction

Injury to the anterior cruciate ligament (ACL) often occurs in contact sports, such as soccer, basketball, and American football (1). Notably, up to 60% of ACL injury events in contact sports involve contact to the trunk and/or upper limbs shortly before or near the estimated time of injury, estimated to be within 100 ms of initial ground contact (IC) (2–4). One of the frequent injury scenarios is the injured player being pushed, pulled, or in contact with external objects/players mid-air, followed by landing on the injured leg with a limitedly flexed and abducted knee position (5–7). The injury situations highlight the importance of understanding how unanticipated mid-air trunk contact alters knee biomechanics during landings. Such investigations may contribute to understanding indirect-contact ACL injury mechanisms, defined as contact with other body parts rather than the injured knee (4), and help players better prepare and respond to unanticipated trunk perturbation.

Trunk motion and perturbation, and their association with ACL injury risk, have gained increased attention recently. ACL injury video analyses have shown that limited trunk flexion (2, 8) and significant lateral trunk bending towards the injured leg (2, 9) are frequently observed in ACL injury events. Such observations are consistent with laboratory investigations of landing biomechanics associated with ACL loading (10–12). For example, active mid-air trunk extension resulted in smaller knee flexion angles, greater peak posterior ground reaction forces (GRF), and greater peak knee extension and adduction moments during double-leg landings, associated with greater ACL loading (10). In contrast, flexing the trunk while landing has been shown to increase knee flexion angles, decrease peak vertical GRF, and decrease peak knee extension moments during single-leg drop landing, associated with decreased ACL loading (13). Meanwhile, mid-air lateral trunk bending resulted in greater peak vertical GRF and greater knee adduction angles of the ipsilateral landing leg relative to the trunk bending direction during double-leg landing (11).

In addition to self-initiated trunk motion, external trunk perturbation also altered lower limb biomechanics associated with ACL loading during landing. Mid-air lateral pulling perturbation in the frontal plane was associated with greater ACL loading of the ipsilateral landing leg during double-leg landing, characterized by greater GRF and smaller knee flexion angles (14). Similarly, mid-air external trunk pushing perturbation increased peak vertical GRF, knee extension moments, and knee adduction moments, while decreasing knee flexion angles for the contralateral leg to the pushing perturbation direction during both double-leg and single-leg landings (15, 16). One recent work quantified trunk perturbation in the sagittal plane, demonstrating that mid-air posterior trunk pulling perturbation reduced trunk and knee flexion angles, elevated peak knee abduction angle, and elevated peak knee extension and adduction moments during double-leg landing (17). Yet, the external pulling perturbation was only applied in one known direction (posterior vs. no perturbation), limiting its relevance to real-world scenarios where perturbations are often unpredictable and occur in multiple planes. Furthermore, double-leg landings were studied in previous investigations, while a large proportion of ACL injuries occur during single-leg landings (6, 18). While previous findings have shown that both trunk motion and perturbations applied in the sagittal or frontal plane altered ACL loading variables during landings (10, 11, 15), the biomechanical effects of multi-plane unanticipated trunk perturbations on single-leg landing biomechanics remain unclear.

The purpose of this study was to determine the effect of the unanticipated external upper-trunk pulling perturbation in the combined posterior and medial-lateral directions on biomechanical variables associated with ACL loading during single-leg landings. Previous studies have shown that contralateral pushing and posterior pulling perturbations applied to the upper trunk were associated with increased ACL loading variables during landings (15, 17). Therefore, the first hypothesis was that both posterior-medial and posterior-lateral pulling perturbations relative to the landing leg would result in increased ACL loading variables, including decreased trunk and knee flexion angles, increased knee abduction angles, and increased knee extension and adduction moments compared to the no-perturbation condition. Additionally, the second hypothesis was that the posterior-lateral perturbation would result in greater increases in those variables associated with greater ACL loading during landing compared to the posterior-medial perturbation.

## 2 Materials and methods

### 2.1 Participants

The reported effect sizes ranged from 0.58 to 1.18 between with and without posterior trunk pulling perturbation for trunk flexion angles and maximal knee extension/adduction moments during double-leg landings in a previous study (17). Based on the smallest effect size of 0.58, a minimum sample size of 26 participants was necessary to detect significant differences with 80% statistical power at an alpha level of 0.05. As previous research has demonstrated consistent responses to external trunk perturbation in males and females (15), the current study recruited participants of both sexes. Thirty-seven injury-free recreational athletes who had experience in jump-landing sports and were physically active at the time of testing were originally recruited in the current study. Data from 33 participants (12 males and 21 females; age: 21.65 ± 2.07 years old; height: 1.7 ± 0.1 m; mass: 68.6 ± 13.7 kg) were utilized for statistical analyses (see Results). Participants were excluded if they had a history of any trunk and lower limb surgery; any major injury resulting in absence from physical activity for more than two weeks in the last six months; or endorsed back pain (10). This study was approved by the University of Kansas Institutional Review Board. Written informed consent was obtained from all participants prior to data collection.

### 2.2 Protocol

Participants changed into spandex clothing and running shoes after signing the informed consent. All participants warmed up through a 3 min self-paced treadmill running and dynamic stretches. Jump height was first assessed using a Vertec (Columbus, OH, USA). Participants were asked to jump with both legs and land on one leg for maximal height. Three separate trials were completed. The landing leg was predetermined and counterbalanced across participants (19). Jump height was calculated as the difference between the average of three jumps and the standing height (16).

**Figure 1:**
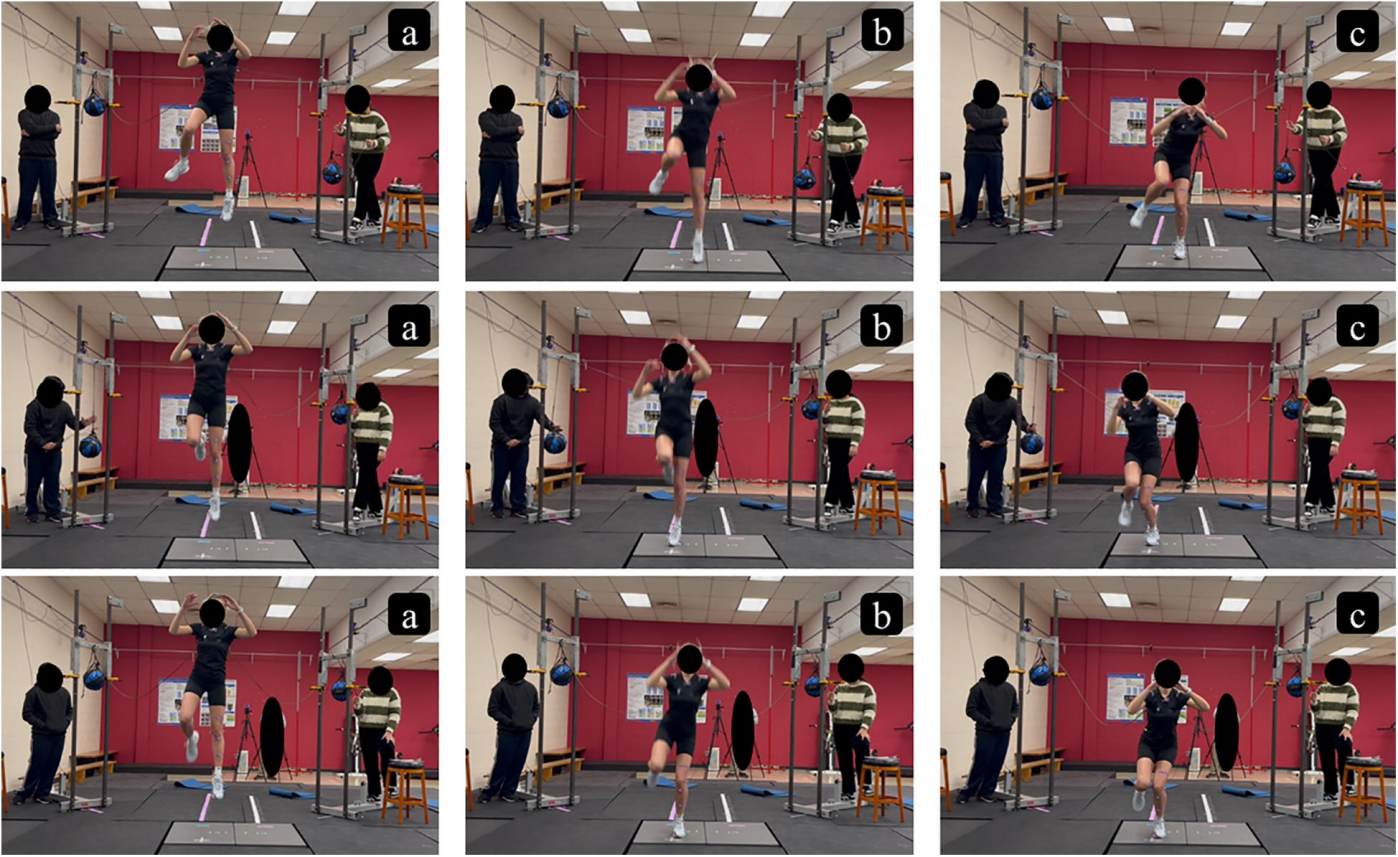
Double-leg jump and single-leg landing at peak jump height **(a)**, initial ground contact **(b)**, and landing **(c)** with posterior-lateral pulling perturbation (top row), posterior-medial pulling perturbation (middle row), and no perturbation (bottom row) applied to the upper trunk.

External trunk pulling perturbations were created using two customized apparatuses (15) positioned 2 meters behind the participant on the posterior-medial and posterior-lateral sides (angled 45° medially and laterally from the participant's midline), respectively (Figure 1). Each apparatus hung a slam ball (4.54 kg) attached to the participant's upper trunk via a strap. The slam ball dropped freely when triggered by a researcher, creating a pulling force (approximately 44.5 Newtons) to the trunk in the corresponding placement direction. Perturbation timing was designed to occur near the peak jump height based on previous studies (15, 16). The perturbation magnitude was selected to be consistent with prior research and to safely induce a moderate perturbation (17). The pulling perturbation mechanism and directions were designed to mimic situations in which an athlete's trunk is restrained by an opponent prior to landing. Examples include cases where the trunk or jersey is held in the air just before the estimated time of injury in soccer, handball, or basketball (2, 7, 9). The 2-meter distance between the apparatus and the participant was determined based on pilot testing to prevent accidental contact with the perturbation apparatuses. The standard strap length was controlled for each side to increase perturbation timing consistency and allow participants' full range of motion during jump-landings.

To familiarize participants with the trunk perturbation and testing conditions, practice trials were performed. Participants first experienced pulling perturbation from each side while standing still to become accustomed to the magnitude. Then, participants completed a minimum of one double-leg jump and single-leg landing under each of three perturbation conditions: (1) no perturbation, (2) posterior-medial pulling perturbation, and (3) posterior-lateral pulling perturbation (Figure 1). Participants initiated the jump with feet shoulder-width apart and were asked to jump as high as possible regardless of perturbation conditions. The perturbation condition was known to the participant during practice trials, and additional practice was allowed if preferred.

After the practice trials, reflective markers were placed on the participant's trunk and the landing leg if they were willing to continue participating after the practice (16). Two additional markers were placed on the diameter of each slam ball to monitor perturbation onset. All marker coordinates were recorded using an eight-camera Vicon motion capture system (Vicon Vero v2.2, Oxford, UK) at a sampling frequency of 120 Hz. GRF was collected through a synchronized force plate (Bertec FP6090-15-TM-2000, Columbus, OH, USA) at a sampling frequency of 1,200 Hz. A static trial was recorded with participants standing still for 5 s. For official trials, a minimum of six successful trials were collected for each perturbation condition (posterior-medial perturbation and posterior-lateral perturbation) and a minimum of three successful trials without perturbation in a randomized order. Participants did not know the perturbation direction prior to each jump. At least a 30 s break was provided between trials to minimize potential fatigue.

A trial was considered successful if it met all of the following criteria: (1) reflective markers on the participant and the slam ball were collected; (2) participant landed with the per-determined leg on the force plate and stood up before lowering the other leg down to the ground; (3) the perturbation occurred within a 100 ms window relative to the peak jump height (15). A trial was repeated if it failed to meet these criteria. The perturbation timing was immediately reviewed by checking the marker positions and counting frames to determine the temporal relationship between the hip markers and the slam ball in the Vicon Nexus software after each jump. The final perturbation timing was later confirmed during data processing using the same 100 ms threshold relative to peak jump height. Overall, a minimum of 15 trials were collected from three conditions during data collection. At the end of the data collection session, participants rated the intensity of the perturbation in relation to their sport experience using a 5-point scale (1 being the minimum intensity and 5 being the maximum intensity) (15) and reported whether they had anticipated the perturbation condition prior to each jump.

### 2.3 Data reduction

Raw kinematic and kinetic data were filtered using a fourth-order Butterworth low-pass filter at 15 Hz for the inverse dynamic approach (20). GRF was separately filtered at 100 Hz for impact force extraction (10). The identification of ankle and knee joint centers, the construction of segment reference frames for the trunk, thigh, shank, and foot, as well as the three-dimensional Cardan angle calculation, followed the details in previous studies (11). The resultant knee joint moments were expressed as internal moments and normalized to the product of body weight and body height. GRF was normalized to body weight.

The perturbation consistency was quantified using perturbation timing, perturbation duration, and perturbation impulse. Perturbation timing was defined as the time interval between the perturbation onset and peak jump height. Positive numbers indicated that the perturbation occurred after peak jump height, whereas negative numbers indicated that it occurred before peak jump height. Perturbation duration was defined as the time from perturbation onset to IC of the landing leg (GRF > 20 N), representing the duration of the mid-air pulling perturbation experienced by the participant. Perturbation onset was identified as the first frame in which the velocity of the slam ball decreased by 10% (17). Perturbation impulse was calculated as the product of perturbation force and perturbation duration based on impulse-momentum theory.

The independent variable was the perturbation condition, including no perturbation, posterior-lateral pulling perturbation, and posterior-medial pulling perturbation relative to the landing leg. Dependent variables associated with ACL loading were calculated at IC and during the early-landing phase (first 100 ms after IC) (10, 11). Kinematic variables included jump height, trunk flexion, and lateral bending (positive number indicating bending toward the landing leg) angles at IC, knee flexion angle at IC, peak trunk flexion and lateral bending angles during early-landing, peak knee flexion, abduction, and internal rotation angles during early-landing. Kinetic variables included peak vertical and posterior GRF during early-landing, peak knee extension, adduction, and external rotation moments during early-landing. All data reduction was performed in MATLAB 2025a (MathWorks, Natick, MA, USA).

### 2.4 Statistical analysis

Trials were excluded from statistical analysis if the perturbation occurred outside the required timing window or if marker data were missing. For each condition, a minimum of two valid trials was retained for every participant. The average of the remaining trials in each condition was then calculated and used for statistical analyses. Paired *t*-tests were performed on perturbation timing, perturbation duration, and perturbation impulse to determine the perturbation consistency between posterior-lateral and posterior-medial pulling perturbations. One-by-three repeated-measures analyses of variances (ANOVAs) were conducted on all dependent variables to identify the effect of perturbation conditions. Paired *t*-tests were also applied if a significant main effect was observed in ANOVAs (alpha level ≤ 0.05). To control the study-wide false discovery rate, the Benjamini–Hochberg procedure was applied to all comparisons at 0.05 (21). Effect sizes for paired comparisons were calculated using Cohen's dz (22). All statistical analyses were performed in SPSS Statistics 22 (IBM Corporation, New York).

## 3 Results

Data from four participants (2 males and 2 females) were excluded from statistical analyses due to missing all trials in at least one condition, caused by missing markers from data collection or perturbation timing that fell outside the required range. In the remaining 33 participants, 64 of the 495 total trials were excluded from analysis for the same reasons. None of the participants reported being able to predict the direction of perturbation during data collection. Participants rated the perceived perturbation intensity as 2.6 ± 0.7 on the 5-point scale, indicating a close-to-moderate perturbation.

No significant differences were found in perturbation timing (Posterior-lateral: 30.90 ± 29.48 ms; Posterior-medial: 20.53 ± 28.48 ms; *p* = 0.813), perturbation duration (Posterior-lateral: 200.66 ± 46.53 ms; Posterior-medial: 207.54 ± 50.97 ms; *p* = 0.276), and perturbation impulse (Posterior-lateral: 8936.7 ± 2072.2 N.ms; Posterior-medial: 9243.2 ± 2270.0 N.ms; *p* = 0.276). Significant perturbation main effects were observed in all dependent variables except jump height and peak knee external rotation moment during early-landing (Table 1). Overall, 41 pairwise comparisons were conducted, and the largest *p*-value was 0.035 following the Benjamini–Hochberg procedure. Effect sizes with corresponding *p*-values for each comparison are shown in Table 2.

**Table 1:** Means ± standard deviations for dependent variables under each perturbation condition and *p*-values of main effects observed in repeated-measures ANOVAs.

Dependent variables (unit)	Posterior-lateral perturbation	Posterior-medial perturbation	No perturbation	*p*-values
Jump height (m)	0.38 ± 0.11	0.38 ± 0.11	0.39 ± 0.11	0.383
Trunk flexion angle at IC (°)	14.30 ± 5.81^b^	11.66 ± 5.65^c^	16.56 ± 5.14^a^	**<0** **.** **001**
Trunk lateral bending angle at IC (°)	13.52 ± 3.03^a^	2.58 ± 2.26^c^	8.52 ± 2.96^b^	**<0**.**001**
Peak trunk flexion angle during early-landing (°)	16.89 ± 7.34^b^	13.21 ± 7.05^c^	20.49 ± 6.79^a^	**<0**.**001**
Peak trunk lateral bending angle during early-landing (°)	17.38 ± 4.73^a^	0.29 ± 2.87^c^	9.80 ± 3.43^b^	**<0**.**001**
Knee flexion angle at IC (°)	10.78 ± 5.26^a^	9.40 ± 5.80^b^	7.89 ± 5.57^c^	**<0**.**001**
Peak knee flexion angle during early-landing (°)	46.41 ± 8.03^a^	43.51 ± 9.93^b^	45.25 ± 8.40^a^	**0**.**001**
Peak knee abduction angle (-) during early-landing (°)	−3.25 ± 2.29^a^	−1.50 ± 2.11^c^	−1.92 ± 1.82^b^	**<0**.**001**
Peak knee internal rotation angle during early-landing (°)	6.91 ± 6.27^a^	4.82 ± 6.45^b^	4.19 ± 6.28^b^	**<0**.**001**
Peak vertical GRF during early-landing (BW)	4.21 ± 0.80^a^	4.02 ± 0.80^b^	4.08 ± 0.89^a^^,^^b^	**0**.**023**
Peak posterior GRF during early-landing (BW)	0.77 ± 0.27^a^	0.67 ± 0.21^b^	0.53 ± 0.23^c^	**<0**.**001**
Peak knee extension moment (-) during early-landing (BW* BH)	−0.122 ± 0.036^a^	−0.131 ± 0.048^a^	−0.109 ± 0.037^b^	**<0**.**001**
Peak knee adduction moment during early-landing (BW* BH)	0.031 ± 0.019^a^	0.019 ± 0.019^b^	0.022 ± 0.018^b^	**0**.**001**
Peak knee external rotation moment (-) during early-landing (BW* BH)	−0.029 ± 0.018	−0.030 ± 0.016	−0.026 ± 0.013	0.495

IC, initial ground contact; GRF, ground reaction force; BW, body weight; BH, body height; ^a^is the greatest, ^b^is the second greatest, and ^c^is the least among perturbation conditions following a significant perturbation main effect observed, while ^a,b^is not significantly differentiated from ^a^ or ^b^. Statistically significant differences are shown in bold.

**Table 2:** Effect size (*p*-value) for dependent variables with a significant perturbation main effect.

Dependent variables	Posterior-lateral vs. no perturbation	Posterior-medial vs. no perturbation	Posterior-lateral vs. Posterior-medial
Trunk flexion angle at IC	**1.00** **(<0.001)**	**1.53** (**<0.001)**	**1.00** (**<0.001)**
Trunk lateral bending angle at IC	**3.16** (**<0.001)**	**1.92** (**<0.001)**	**3.16** (**<0.001)**
Peak trunk flexion angle during early-landing	**1.19** (**<0.001)**	**2.14** (**<0.001)**	**1.19** (**<0.001)**
Peak trunk lateral bending angle during early-landing	**3.15** (**<0.001)**	**2.45** (**<0.001)**	**3.15** (**<0.001)**
Knee flexion angle at IC	**0.56** (**<0.001)**	**0.53** (**0.004)**	**0.56** (**0.003)**
Peak knee flexion angle during early-landing	0.66 (0.043)	**0.38** (**0.035)**	**0.66** (**0.001)**
Peak knee abduction angle during early-landing	**1.35** (**<0.001)**	**0.47** (**0.011)**	**1.35** (**<0.001)**
Peak knee internal rotation angle during early-landing	**0.56** (**<0.001)**	0.18 (0.316)	**0.56** (**0.003)**
Peak vertical GRF during early-landing	0.48 (0.092)	0.18 (0.307)	**0.48** (**0.009)**
Peak posterior GRF during early-landing	**0.60** (**<0.001)**	**1.06** (**<0.001)**	**0.60** (**0.002)**
Peak knee extension moment during early-landing	**0.35** (**<0.001)**	**1.00** (**<0.001**)	0.35 (0.051)
Peak knee adduction moment during early-landing	**0.59** (**<0.001)**	0.18 (0.300)	**0.59** (**0.002)**

IC, initial ground contact; GRF, ground reaction force; BW, body weight; BH, body height. Statistically significant differences are shown in bold.

Both conditions with perturbation resulted in significantly smaller trunk flexion angles at IC, smaller peak trunk flexion angles, greater knee flexion angles at IC, greater peak posterior GRF, and greater peak knee extension moments during early-landing compared to the no perturbation condition (Table 1). Posterior-lateral perturbation resulted in the greatest trunk lateral bending angles at IC and at peak, knee flexion angles at IC, peak knee abduction and internal rotation angles, peak posterior GRF, and peak knee adduction moments during early-landing among the three conditions. Posterior-medial perturbation demonstrated the least trunk flexion angles at IC and at peak, trunk lateral bending at IC and at peak, peak knee flexion angles, and peak knee abduction angles during early-landing.

## 4 Discussion

The current study aimed to quantify the influence of unanticipated trunk pulling perturbation in the combined posterior and medial-lateral directions on landing biomechanics associated with ACL loading and injury risk during single-leg landings. The perturbations were consistent between posterior-lateral and posterior-medial directions, and jump height was consistent across the two perturbation and no-perturbation conditions. Perturbation occurred approximately 20–30 ms after the peak jump height, leading to about 200 ms of pulling force applied prior to landing. Overall, the current perturbation magnitude was similar to previous studies (15–17), indicating a close-to-moderate objective intensity relative to real-game contacts. It should be noted, however, that while perturbation timing was well controlled, the exact intensity was not identical for every participant and should be considered when interpreting the results.

The results generally supported the first hypothesis that unanticipated trunk perturbation (both posterior-lateral and posterior-medial directions) would result in increased ACL loading variables compared to the no perturbation condition, demonstrated by decreased trunk flexion angles, increased peak posterior GRF, and increased peak knee extension moments during early-landing. Yet, the perturbations also resulted in greater knee flexion angles at IC, which did not support the first hypothesis. Both perturbations applied a posterior force component to the upper trunk, which may have generated an external trunk extension moment to resist trunk flexion, given that the upper trunk is located superior to the center of mass (11, 15). As such, trunk flexion was reduced both at IC and throughout early-landing. The restricted trunk flexion likely increased the horizontal distance from the trunk center of mass to the knee joint during early-landing, leading to increased knee extension moments, which were associated with increased ACL loading in the sagittal plane (11, 13). These findings aligned with previous studies, showing that self-initiated trunk extension and external posterior trunk pulling perturbation both resulted in greater ACL loading variables in the sagittal plane during landings (11, 17). Compared to earlier studies that focused on double-leg landings (15, 17), the current single-leg landing pattern demonstrated significantly greater magnitude of peak GRF and knee moments and smaller knee flexion angles, indicating a greater risk for ACL injuries and supporting the common single-leg landing scenarios observed in ACL injury events (2, 23). However, the greater knee flexion angle at IC was observed when the perturbation was applied, which was associated with decreased ACL loading during landing. The increased knee flexion angles at IC could be a compensatory strategy to rely more on the knee joint to dissipate the landing forces while the trunk flexion was limited. However, the increased knee flexion angles did not necessarily increase the knee flexion range of motion or decrease landing forces. Actually, the knee flexion range of motion was likely decreased for the perturbation conditions, as their peak knee flexion angles were similar to those of the no perturbation conditions. Overall, these results suggest that unanticipated pulling perturbations limited trunk flexion and resulted in increased knee moments associated with increased ACL loading in the sagittal plane during single-leg landing.

The findings partially supported the second hypothesis as the posterior-lateral pulling perturbation demonstrated the greatest trunk lateral bending angles, peak knee abduction and internal rotation angles, peak posterior GRF, and peak knee adduction moments during landing, associated with increased ACL loading variables in the frontal plane. In contrast, the posterior-medial perturbation resulted in smaller trunk and knee flexion angles at IC and at peak values during landing compared to the posterior-lateral perturbation, which did not support the second hypothesis. The posterior-lateral external force applied through the pulling perturbation imposed a lateral force component. Because this force was applied superior to the pelvis, it likely generated an external lateral trunk bending effect. Given that the trunk occupied a significant portion of body weight (24), extensive lateral trunk bending shifted the whole-body center of mass laterally relative to the base of support. This mechanical misalignment likely contributed to the observed increase in knee abduction and internal rotation angles, as well as internal knee adduction moments. The current results were consistent with previous studies, which have shown that similar mechanical effects caused by ipsilateral pulling (14) or pushing perturbation (15, 16) result in increased ACL loading variables in the frontal plane. On the other hand, posterior-medial perturbation showed the smallest trunk flexion angles at IC and peak values, the smallest peak knee flexion angles among all conditions, associated with increased ACL loading in the sagittal plane. Greater peak posterior GRF and knee extension moments were also observed in the posterior-medial perturbation direction compared to no perturbation. These sagittal-plane mechanics are associated with increased ACL injury risk factors (6, 25, 26). Although the posterior–medial perturbation also applied an external force, it produced limited trunk lateral bending angles compared to other conditions. This may be because excessive bending of the trunk away from the landing leg would compromise stability, as participants were instructed to maintain balance with the landing leg serving as the sole base of support. Therefore, the medial pulling force pulled the trunk into a more neutral posture. However, the restricted trunk flexion angle likely shifted the trunk center of mass posteriorly relative to the base of support, which likely decreased stability and limited the peak knee flexion angles during landing. Overall, the posterior-medial perturbation resulted in increased ACL loading variables in the sagittal plane during single-leg landing.

The findings of this study provided practical implications. Unanticipated upper trunk perturbations in the combined posterior and medial-lateral direction altered trunk control and increased knee loading during single-leg landing. Importantly, these changes in knee biomechanics were observed even though the perturbation applied in mid-air was evaluated as moderate intensity, highlighting the impact of external trunk forces on landing mechanics. More specifically, posterior-lateral pulling perturbation increased ACL loading in both sagittal and frontal planes through limited trunk flexion and increased lateral bending, while posterior-medial perturbation primarily elevated sagittal plane loading by restricting trunk and knee flexion. Such information contributes to a better understanding of indirect-contact ACL injury mechanisms. Future video analyses of ACL injury events need to incorporate the perturbation mechanism (pulling vs. pushing), direction (anterior-posterior or medial-lateral), timing (mid-air, takeoff, or landing), and trunk posture both before and after landing to better quantify ACL injury characteristics. Training in dynamic and unpredictable environments, such as with resistance band-induced perturbations, may provide opportunities to improve athletes' awareness under real-world playing situations. However, such training should be carefully designed and implemented by qualified athletic trainers to ensure safety and effectiveness. Coaches and practitioners may also consider active trunk flexion and neutral frontal plane trunk posture during landing drills. It should be noted that the efficacy of such approaches has not yet been established and should be evaluated in future intervention studies.

Several limitations persisted in the current study. First, although pulling perturbation is feasible and has been consistently controlled in the current study to simulate dynamic contacts, it may not closely mimic real-world contact events (e.g., collisions with opponents). Yet, given the challenge of applying unpredictable pushing perturbations safely involving the sagittal plane, the current approach is likely an appropriate surrogate in laboratory investigations. Second, a fixed perturbation magnitude was used for all participants. While perceived intensity was rated consistently, individuals with different body masses may have experienced different biomechanical responses. Additionally, although no significant differences in perturbation timing and impulse were observed across conditions, the exact intensity was not identical for every participant. Future studies need to investigate the effects of graded perturbation magnitudes in relation to body size and strength on landing mechanics. Lastly, the sample size was unbalanced between sexes, with more females than males included. Although the sex differences in ACL injury risk are well-documented (27–29), previous findings showed similar responses across perturbation conditions in both males and females (15). Therefore, while the sex imbalance limits the ability to make direct comparisons between groups, it is unlikely to have substantially influenced the overall findings of this study.

## 5 Conclusion

Unanticipated upper trunk pulling perturbations in the combined posterior and medial-lateral directions significantly altered landing biomechanics associated with ACL loading during single-leg landings. Specifically, posterior-lateral perturbation resulted in increased trunk lateral bending, increased knee abduction and internal rotation angles, elevated peak posterior GRF, and greater knee joint moments during landing. Additionally, posterior-medial perturbation primarily limited trunk and knee flexion and increased peak posterior GRF and knee extension moments during landing. These findings contribute to the understanding of indirect-contact ACL injury mechanisms and provide evidence linking unanticipated trunk perturbations to altered knee joint loading patterns during single-leg landing.

## Data Availability

The raw data supporting the conclusions of this article will be made available by the authors, without undue reservation.
